# Continuous control of tracheal cuff pressure and microaspiration of gastric contents: a randomized controlled study

**DOI:** 10.1186/cc9578

**Published:** 2011-03-11

**Authors:** S Nseir, F Zerimech, C Fournier, R Lubret, P Ramon, A Durocher, M Balduyck

**Affiliations:** 1 University Hospital, Lille, France

## Introduction

Underinflation of a tracheal cuff frequently occurs in critically ill patients, and results in microaspiration of contaminated oropharyngeal secretions and gastric contents that plays a major role in the pathogenesis of VAP. The aim of this study was to determine the impact of continuous control of cuff pressure (P_cuff _) on microaspiration of gastric contents.

## Methods

Patients requiring mechanical ventilation through a PVC-cuffed tracheal tube >48 hours were eligible. Patients were randomly allocated to continuous control of P_cuff _using a pneumatic device (Nosten^®^) (intervention group, *n *= 61) or routine care of P_cuff _(control group, *n *= 61). Target P_cuff _was 25 cmH_2_O in the two groups. The primary outcome was microaspiration of gastric contents as defined by the presence of pepsin at a significant level (> 200 ng/ml) in tracheal secretions. Secondary outcomes included incidence of microbiologically confirmed VAP (tracheal aspirate >10^5 ^cfu/ml), incidence of tracheobronchial colonization, and tracheal ischemic lesions as defined by a macroscopic score. Pepsin was quantitatively measured in all tracheal aspirates during the 48 hours following randomization. A patient was considered as having abundant microaspiration when >65% of tracheal aspirates were pepsin positive. Patients remained in a semirecumbent position in bed, and a written enteral nutrition protocol was used. All analyses were performed on an intention-to-treat basis.

## Results

Patient characteristics were similar in the two groups. The pneumatic device was efficient in controlling P_cuff_. Pepsin was measured in 1,205 tracheal aspirates. The percentage of patients with abundant microaspiration (18% vs. 46%, *P *= 0.002, OR (95% CI) 0.25 (0.11 to 0.59)), pepsin level (median (IQ) 195 (95 to 250) vs. 251 (130 to 390), *P *= 0.043), and VAP rate (9.8% vs. 26.2%, *P *= 0.032, 0.30 (0.11 to 0.84)) were significantly lower in the intervention group compared with control group. However, no significant difference was found in rate of patients with tracheobronchial colonization (34% vs. 39%, *P *= 0.7) or in tracheal ischemia score (4.5 (1 to 6) vs. 4.5 (1 to 7), *P *= 0.9) between the two groups.

## Conclusions

Continuous control of P_cuff _is associated with significantly decreased microaspiration of gastric contents in critically ill patients.

